# Effects of Soy Isoflavones on Biochemical Markers of Bone Metabolism in Postmenopausal Women: A Systematic Review and Meta-Analysis of Randomized Controlled Trials

**DOI:** 10.3390/ijerph18105346

**Published:** 2021-05-17

**Authors:** Wiesław Kanadys, Agnieszka Barańska, Agata Błaszczuk, Małgorzata Polz-Dacewicz, Bartłomiej Drop, Maria Malm, Krzysztof Kanecki

**Affiliations:** 1 Specialistic Medical Center “Czechów” in Lublin, 20-848 Lublin, Poland; wieslaw.kanadys@wp.pl; 2 Department of Medical Informatics and Statistics with E-learning Lab, Medical University of Lublin, 20-090 Lublin, Poland; bartlomiej.drop@umlub.pl (B.D.); maria.malm@umlub.pl (M.M.); 3 Department of Virology with SARS Laboratory, Medical University of Lublin, 20-093 Lublin, Poland; agata.blaszczuk@umlub.pl (A.B.); malgorzata.polz.dacewicz@umlub.pl (M.P.-D.); 4 Department of Social Medicine and Public Health, Warsaw Medical University, 02-007 Warsaw, Poland; kkanecki@wum.edu.pl

**Keywords:** soy isoflavones, natural products, bone turnover markers, postmenopausal women, bone mineral density

## Abstract

This systematic review and meta-analysis of randomized controlled trials was performed to more completely assess potential changes in bone turnover marker levels in postmenopausal women during the intake of soy isoflavones. PubMed (Medline) and EMBASE were searched for relevant studies, and their quality was evaluated according to Cochrane criteria. The levels of markers were evaluated in a total of 1114 women who ingested mean daily doses of 98.2 mg (30.9 to 300) of soy isoflavones for 3 to 24 months, in comparison to those of 1081 subjects who used a placebo. Ten, eighteen, eight, and fourteen comparison studies were finally selected for an estimation of the effects on osteocalcin (OC), bone alkaline phosphatase (BAP), pyridinoline (PYD), and deoxypyridinoline (DPD), respectively. A summary of the results of intervention was as follows: 4.16%, 95% CI: −7.72–16.04, *p* = 0.49 for OC; 5.50%, 95% CI: −3.81–14.82, *p* = 0.25 for BAP; −12.09%, 95% CI: −25.37–1.20, *p* = 0.07 for PYD; and −7.48%, 95% CI: −15.37–0.41, *p* = 0.06 for DPD. The meta-analysis of the included studies revealed some statistically insignificant observations that soy isoflavones intake is associated with a trend in increased levels of OC and BAP, as well as a trend in reduced levels of PYD and DPD. Soy isoflavones may have a beneficial effect on bone formation markers, but this requires extensive multi-center research.

## 1. Introduction

The human skeleton continually adjusts to ever-changing biomechanical conditions [1]. Bone metabolism consists of two main processes: bone building (modeling)—which lasts throughout the entire growth period until puberty—and bone remodeling, which goes on throughout life. These processes cooperate in the growing skeleton to define the appropriate skeletal shape, to maintain proper serum levels of ions, and to repair structurally compromised regions of bone [2]. In adults, bone tissue undergoes continuous remodeling by the coordination and balance between processes of resorption and formation through osteoclasts and osteoblasts activity, at sites called ‘bone multicellular units’ (BMUs) [3,4]. This allows a bone to adapt to changes in mechanical load, provides repair of microdamages or fractures, and is a mechanism for its 3–6-month renewal via bone resorption, formation, and mineralization [5]. The dynamics of bone growth and loss depend on age. Bone accretion occurs from birth, lasts throughout childhood and adolescence, and reaches individual maximum bone mass between 16 and 30 years of age, depending on the type of bone tissue and the location in the skeleton [6,7,8]. Although bone mass is mainly genetically determined, many factors affect its quality, including hormone economy, physical activity, eating habits, and stimulants [9]. Bone value in later life depends on both the peak bone mass and the rate at which it is lost [10]. After a period of relative stabilization, towards the end of the fourth decade of life, bone tissue is gradually lost at a rate of 0.3–0.5% per year at most skeletal sites. This is an expression of the increasing domination of the resorption process over that of bone formation [11]. During the menopausal transition, a decline in ovarian activity and accompanying estrogen deficiency and increased follicle stimulating hormone (FSH), which overlaps with age-related changes, results in significantly increased bone turnover [12,13,14]. Bone loss increases two years before menopause and is at its highest level during the first 3 years of menopause, with losses reaching 3–5% annually [15]. After 5–10 years, a slow phase is observed that lasts indefinitely. The level of bone loss is, respectively, that of 0.6%, 1.1%, and 2.1% per year for the 60–69, 70–79, and >80 age groups [16,17].

Various biomolecules, so-called ‘bone turnover markers’, reflect the metabolic activity of bone tissue. These are fragments of bone structural protein components (or their degradation products) and enzymes released into bloodstream during the metabolic activity of osteoblasts and osteoclasts. Changes in bone marker synthesis take place after 3 to 6 months [18]. The examination of markers from either group in serum or urine provides an insight into the dynamics of bone turnover. Such work fairly rapidly reveals early changes in resorption or formation, and thereby complements the much slower assessment of morphologic changes by bone density measurements. Bone markers can also be used for a quick evaluation of the effects of hormonal replacement therapy (HRT) [19]. Prospective observational studies have shown that bone loss during menopause is reflected in changes in bone turnover markers, as well as in markers of both bone resorption and formation increase during menopausal transition [20,21].

In the menopausal period, hot flashes, sleep disturbances, and other menopausal symptoms are predominant, including osteoporotic disorders. Osteoporosis brings about the most important long-term effects and seriously impacts the quality of life of postmenopausal women [22]. This can be mitigated to some degree by HRT. However, many women refuse HRT for a variety of reasons, including the fear of cancer and because of adverse effects such as weight gain. Hence, women often demand non-hormonal treatment, including nutraceuticals—pharmaceutical alternatives with medicinal properties, extracted from food or plants [23]. Numerous epidemiological studies and related meta-analyses suggest that soy consumption may be associated with a bone turnover [24]. Special attention is paid to isoflavones (Isof)—phenolic compounds of soybeans that have numerous health-promoting properties. Their structural similarity to 17-β-estradiol allows them to induce estrogenic and antiestrogenic effects by binding to estrogen receptors, and their consumption has been associated with bone metabolism [25].

This systematic review and meta-analysis was performed to provide an assessment of potential changes in the value of biochemical markers of bone turnover in postmenopausal women taking soy protein and/or isoflavones, and is an attempt to determine the factors determining the response of markers during treatment.

## 2. Materials and Methods

### 2.1. Search Strategy and Study Selection

PubMed (Medline) and EMBASE were searched from the year 2000 up to June 2020 to identify works investigating the effect of soy isoflavones on markers of bone turnover [26]. The search process was independently carried out by a minimum of two investigators, and all screening conflicts were solved by consensus throughout the research team. The following word search terms in various combinations were used: ‘randomized controlled trials’ AND ‘postmenopause’, or ‘menopause’ AND ‘soy isoflavones’, or ‘soy protein’ AND ‘bone’, or ‘bone turnover markers’, or ‘bone resorption markers’, or ‘bone formation markers’.

Articles were initially evaluated according to title and/or abstract. Next, the decision was made whether to include or exclude, after an independent and double analysis of full texts of the elected works. Additional article analysis was accomplished by searching references lists of selected (relevant) studies or systematic reviews related to the topic of the work. Inclusion criteria were that the study was of parallel-arm or cross-over design, it contained data for the first period, its follow-up period was at least 3 months, the subjects were postmenopausal women, and it involved a daily amount of soy protein with/or Isof—with clearly described composition of isoflavones and their doses. Moreover, it included a control group (i.e., placebo or protein not containing Isof), a baseline and final of means, with SD values of each bone marker mentioned above (OC, BAP, procollagen type I amino-terminal propeptide (PINP), procollagen type I carboxy-terminal propeptide (PICP), PYD, DPD, and amino-terminal cross-linking telopeptide of type I collagen (NTx), CTX) in the active and control groups. The exclusion criteria were that the study had men and women or premenopausal women as participants, there were insufficient quantitative data, the results were reported as graphics, and the study duration was less than 12 weeks. In addition, exclusion occurred if Isof was mixed with other active formulations, such as calcium, Vitamin D, or Vitamin B12, or there were duplicate reports and the articles were not published in English.

### 2.2. Data Extraction

Data were extracted by the lead author and subsequently reviewed by the co-authors for accuracy. From each of the included studies, the following data were abstracted: first author’s name, year of publication, country of origin, study design; follow-up period, number of participants in intervention and control group; characteristics of the studied populations: age (range), menopause status (years since menopause), body mass index; daily dose of soy protein and Isof (composition of Isof); type of control group; and mean baseline and end-point values for markers of bone turnover, along with the corresponding standard deviation (SD). Where the statistical information was reported in 95% confidence intervals (CI) or standard error (SE), this was converted to SD using appropriate formulas. To avoid duplication of data of measurements of bone markers in trials with multiple time points, only the results from the longest follow-up were taken into account. In the case of trials with more than one active group (different content of Isof), compared to one control group, both results were taken into account; similarly, the data reported in one multicenter international study were analyzed for each center separately.

### 2.3. Quality Assessment and Bias Risk of the Trials

The quality of trials was evaluated using the Cochrane Collaboration tool. This lists seven items that have a potential biasing influence on the estimates of intervention effectiveness in randomized studies, and includes: random sequence generation, allocation concealment, blinding of participants and personnel, blinding of outcome assessment, attrition bias (incomplete outcome data), reporting bias (selective reporting), and other sources of bias. The risks of bias in RCTs are designated in the review as ‘High risk’, ‘Unclear’, or ‘Low risk’ [27]. To explain the possible presence of publication bias, Begg’s test (a rank correlation method based on Kendall’s tau) and Egger’s test (a linear regression method) were applied [28,29]. We also checked for funnel plot symmetry. Here, in the absence of bias, the plots will resemble a symmetrical funnel, as the results of minor studies will scatter at the left side of the plot and the spread will narrow among the major studies on the right side of the plot [30].

### 2.4. Statistical Analysis and Meta-Analysis

As different units of concentration of bone turnover markers were used in the analyzed studies, for comparison of the general effect of soy Isof on these markers, we adopted a method of assessing changes in relation to the initial values in the form of a percentage. Data on individual markers in both the intervention and control groups were presented as number of subjects (*n*) and percentage difference in means (MD) [(final value − initial value) ÷ initial value × 100%]. None of the studies reported sufficient information, however, to allow us to directly calculate the variance of change between pre- and post-intervention values; hence, the missing SDs of MDs were derived using the methods set out in the Cochrane Handbook for Systematic Reviews of Interventions [27]. As suggested by Follman et al. [31], we assumed a correlation coefficient of 0.5. The SDs for percentage change were then calculated by dividing SD for change by baseline value.

Weighted mean difference (WMD) was calculated by subtracting the percentage difference in mean between the control and active groups. A random-effects model was employed for derivation, and 95% CI, and *p* < 0.05 was considered statistically significant [32]. We utilized STATISTICA Medical Software (StatSoft, Krakow, Poland) for all statistical analyses.

In evaluating heterogeneity, Cochrane’s Q and *I*^2^ statistic were applied. The *I*^2^ test allowed us to assess whether the variance cross studies was correct and not due to a sampling error. Furthermore, the degree of heterogeneity was indicated in calculating the percentage of total variation. Here, *I*^2^ values of ≤25% were considered low, >25% as moderate, and ≥75% as high heterogeneity [33].

### 2.5. Subgroup Analysis

An additional subgroup analysis was undertaken in order to detect sources of heterogeneity according to the participant covariate variables. These included: age of participants: <54 year vs. ≥54 year; follow-up period: ≤6 months vs. >6 months; body mass index (BMI): ≤24.9 kg/m^2^ (normal weight) vs. >24.9 kg/m^2^ (overweight and obesity); postmenopausal status: early (<5 year) vs. late (≥5 year); supplement types: soy Isof extract vs. soy protein containing Isof (dietary Isof); and the total dose of Isof per day: <80 mg vs. ≥80 mg. To explore the possible influence of abovementioned covariates and determine the possible impact of Isof on individual variables, we also performed a meta-regression [34,35].

## 3. Results

**Figure 1:**
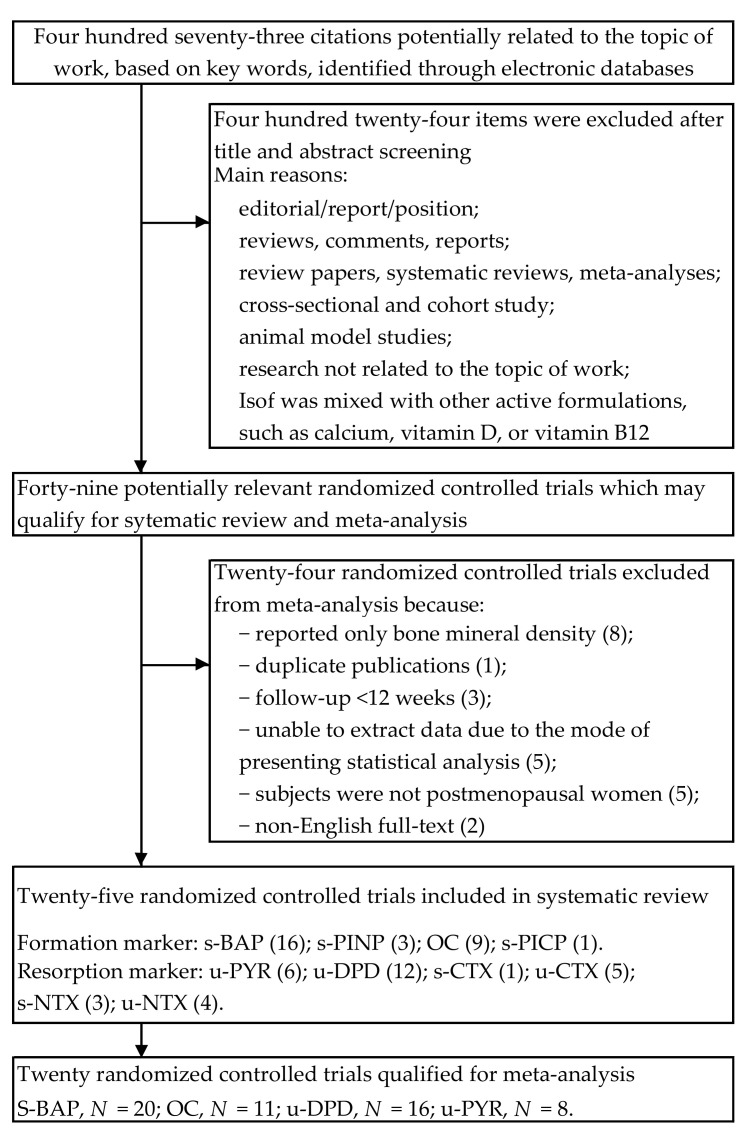
Flow diagram of literature search and research selection procedure. *N*, number of comparisons; (), number of trials; s, serum; u, urine; ALP, total alkaline phosphatase; BAP, bone alkaline phosphatase; CTX, C-terminal telopeptides of type 1 collagen; DPD, deoxypyridinoline; NTX, N-terminal telopeptide of type 1 collagen; OC, osteocalcin; PICP, C-terminal propeptides of type 1 collagen; PINP, N-terminal propeptides of type 1 collagen; PYD, pyridinoline.

A detailed review of our selection procedures is shown in Figure 1. As a result of the search of electronic databases, 473 citations were identified. Titles and abstracts were checked in the initial selection phase, and 427 items were excluded due to irrelevance. In the second phase, 46 articles with potentially significant randomized controlled trials were identified and submitted for full-text assessment. Of these, 21 papers contained duplicate publications, only bone mineral density was reported, the required data were missing, or they did not meet all the inclusion criteria. Therefore, a total of twenty-three studies were included in our final analysis [36,37,38,39,40,41,42,43,44,45,46,47,48,49,50,51,52,53,54,55,56,57,58].

### 3.1. Characteristics of Included Trials

The characteristics of the selected studies are reported in Table 1. Included trials were published between 2001 and 2020. Eight studies were conducted in North America [38,41,44,45,48,52,53,54], seven in Asia [40,46,47,51,55,56,58], six in Europe [36,37,42,43,50,57], one in Australia [39], and one was an international survey (Netherlands, Italy, France) [49]. In nine trials [38,39,41,42,44,45,48,51,57], the primary outcomes were assessments of the effects of soy Isof on markers of bone turnover, while the investigations reported in the other studies led to other main outcomes (e.g., effect on bone mineral density, menopausal symptoms relief), and their secondary result was the assessment of changes in the level of bone markers after of Isof supplementation (which was the subject of our interest). The study design of all included trials involved parallel [36,37,38,39,40,41,43,45,46,47,48,49,50,51,52,53,54,55,56,57,58] and cross-over groups [42,44]. The follow-up of these studies varied widely, ranging from 3 months [36,38,39,40,42,56] to 24 months [50,53,55], including one study of 4 months’ duration [41], seven studies of 6 months [44,46,47,51,57,58], one study of 9 months [48], and five studies with a duration of 12 months [37,43,45,49,52]. Data were pooled from studies comprising a total of 2198 postmenopausal women, including 1122 and 1076 participants in the intervention and placebo arms (individuals of the cross-over trials were considered as treatment and control), respectively. The average age was 56.9 years (median, 54.3; range, 49.2–72.9). In nine of these studies [36,38,39,41,43,45,48,52,57], soy protein containing Isof was used, and soy Isof tablet administration was assessed in other studies. Isof intake varied from 30.9 to 300 mg/day in various treatment groups, and the total mean dose was 98.2 mg (median, 90 mg). Soy Isof supplements were administrated as a drink mix, a powder to mix with food, and tablets. In studies using soy protein containing Isof, its average intake was 29.9 g/d (15–60 g/d). Control groups received casein [36,39], milk protein [38,43,48,53], soy-free protein blend [41,45,52], and soy protein without Isof content [57], or tablets as a placebo.

### 3.2. Assessment of Study Quality

**Figure 2:**
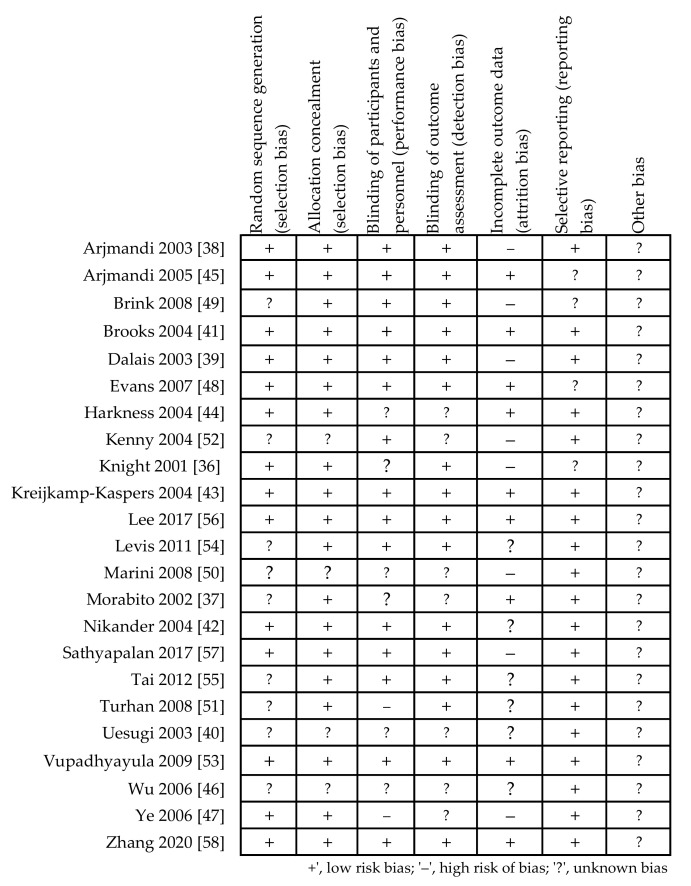
Summary of Cochrane risk of bias of methodological quality for each covered study.

**Figure 3:**
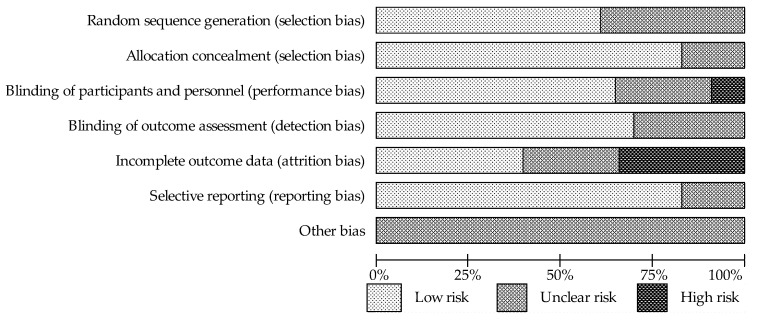
The summary of authors’ evaluation of each category of Cochrane risk bias for all included studies in percent.

The quality of the included studies was evaluated according to instructions listed in the Cochrane Hand Book for Systematic Reviews of Interventions, based on a risk of bias summary for each study (Figure 2) and a risk of bias for each item (Figure 3). The vast majority of the evaluated trials showed a low-risk bias for incomplete outcome data and for selective outcome reporting. It was possible to observe that the studies presented the most unclear risk bias in relation to the blinding (single blinding was recognized in two studies and considered as a high risk). Moreover, thirty-five percent of the trials had risk of bias due to high attrition (dropout > 20%) caused by intolerance to soy bar, irregular self-administration of the investigated drugs, gastrointestinal disturbances, lack of effect, those who started hormone replacement therapy, or compliance with the study procedures. This outcome is likely due to the long duration of the interventions for bone protection against osteoporosis.

### 3.3. Association between Soy Isoflavones Supplementation and Bone Turnover Markers

A meta-analysis was performed regarding the interventions undertaken, since some studies had more than one intervention group; thus, a total of twenty-six interventions were analyzed from twenty-three randomized controlled trials assessing the influence of soy protein Isof or of soy Isof extracts on individual bone turn-over markers.

**Figure 4:**
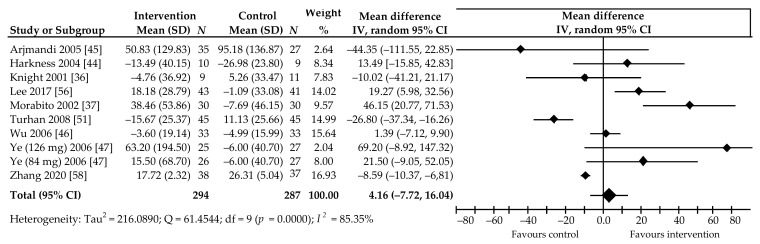
Percentage change in serum Osteocalcin values associated with intake of soy isoflavones, compared with placebo. Note: numbers following authors’ name indicate Isof dose in studies with more than one treatment arm; data calculated from the random-effects model are presented as weighted mean difference; IV, inverse variance; df, degrees of freedom; the horizontal lines denote the 95% CIs, some of which extend beyond the limits of the scale.

Ten comparisons of data from nine trials [36,37,44,45,46,47,51,56,58], in which 581 postmenopausal women participated, contributed to the meta-analysis we applied to the effects of soy Isof on OC level (Figure 4). Compared to control group, a percentage increase in the OC level was noted in six comparisons, including two in which the changes were statistically significant [37,56]; three comparisons indicated an insignificant decrease in marker level, and there were two wherein the reduction was significant [51,58]. Intake of the average daily dose of 85.3 (54–134.4) mg Isof for a period of 3–12 months was not associated with a significant percentage increase in marker level by 4.16%, 95% CI: −7.72 to 16.04, *p* = 0.49, with relatively high heterogeneity (*I*^2^ = 85.35%) (Figure 4).

The result of applying Begg’s test (Kendall’s Tau with continuity correction = −0.52, z = −1.65, *p* = 0.10), as well as the result of utilizing Egger’s test (intercept = 1.40, 95% CI: −0.71 to 3.52, t = 1.53, *p* = 0.16) indicate a lack of evidence of publication bias. Furthermore, subgroup analyses based on the aforementioned pre-specified factors did not reveal significant effects on OC (Table 2). Multivariable meta-regression with the covariate of supplement type (β = −59.83, 95% CI: −118.86 to −0.80, *p* = 0.04) showed this covariate had a significant impact on OC value. However, the age of the participants (β = −0.13, 95% CI: −28.06 to 7.80, *p* = 0.27), follow-up period (β = −10.13, 95% CI: −28.06 to 7.80, *p* = 0.27), BMI (β = 48.92, 95% CI: −13.57 to 111.41, *p* = 0.125), postmenopausal status (β = 12.31, 95% CI: −16.7 to 41.34, *p* = 0.41), and dose of Isof (β = −3.48, 95% CI: −34.42 to 27.46, *p* = 0.82) were found to have no significant influence on these markers.

**Figure 5:**
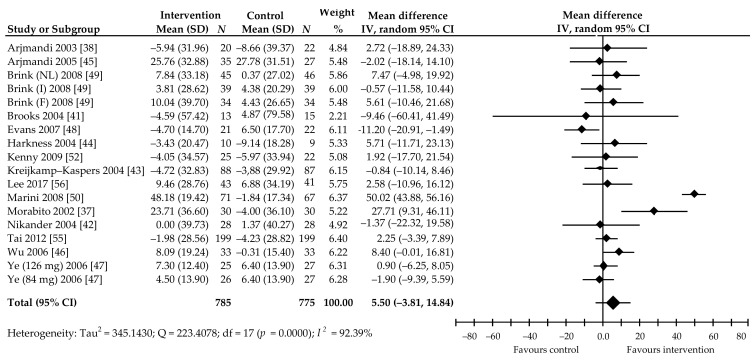
Percentage change in serum BSAP values associated with intake of soy isoflavones compared with placebo. Note: after the author’s name, Isof dose in studies with more than one treatment arm, or abbreviations of the names of the countries where the study was conducted (NL, the Netherlands; I, Italy; F, France), are revealed; data calculated from the random-effects model are presented as weighted mean difference; IV, inverse variance; df, degrees of freedom; the horizontal lines denote the 95% CIs.

In turn, the 18 comparisons from 15 trials [37,38,41,42,43,44,45,46,47,48,49,50,52,56,58] focused on assessing the effect of soy Isof on BAP concentrations in 1533 women receiving a mean dose of 99 (41.9–300) mg Isof per day over a period of 3–24 months. Of these, 11 comparisons showed a percentage increase in the marker level in the treated group, including two with a statistically significant increase [37,50] compared to the corresponding control group; six showed a slight decrease and one was a significant decrease [48]. The random-effects meta-analysis also showed non-significant increase in marker level. Pooled mean percentage change was 5.50%, 95% CI: −3.81 to 14.82, *p* = 0.25 (Figure 5).

The major problem indicated by this analysis is the large heterogeneity of effect of soy Isof (*I*^2^ = 92.39%). However, Begg’s test (Kendall’s Tau with continuity correction = 0.18, z = 0.82, *p* = 0.41) and Egger’s test (intercept = −1.86, 95% CI: −6.02 to 2.30), t = −0.95, *p* = 0.36) did not show evidence of publication bias. Moreover, subgroup analyses based on the abovementioned pre-specified factors did not reveal subgroups with significant effects on BAP (Table 2). In contrast, multivariable meta-regression with the covariates of age of participants (β = −17.07, 95% CI: −25.54 to −8.60, *p* < 0.001), follow-up period (β = −16.10, 95% CI: −23.88 to −8.32, *p* < 0.001), BMI (β = 15.54, 95% CI: 0.41–30.68, *p* = 0.04), dose of Isof per day (β = 19.87, 95% CI: 13.02–26.72, *p* < 0.001), and supplement type (β = 12.63, 95% CI: 3.07–22.20, *p* = 0.01) revealed that these covariates had a significant impact on BAP. Postmenopausal status (β = −6.94, 95% CI: −19.14 to 5.25, *p* = 0.26) was found to have no significant influence on this marker.

**Figure 6:**
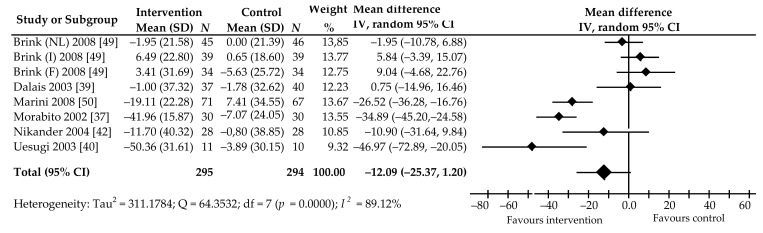
Percentage change in urine PYD values associated with intake of soy isoflavones compared with placebo. Note: abbreviations in parentheses following the author’s name indicate the name of the countries in which the research was conducted (NL, Netherlands; I, Italy; F, France); data calculated from the random-effects model are presented as weighted mean difference; IV, inverse variance; df, degrees of freedom; the horizontal lines denote the 95% CIs.

Changes in the level of PYD in 589 post-menopausal women after the intake of soy Isof in a dose of 81.2 (30.9–118) mg per day for 3–24 months was evaluated in six trials (eight comparisons) [37,39,40,42,49,50]. Among these, five comparisons showed a percentage decrease in PYD value, of which the reduction in three studies [37,40,50] was statistically significant compared to control group. An insignificant increase was noted in three comparisons. The pooled mean percentage change was −12.09%, 95% CI: −25.37 to 1.20, *p* = 0.07, with high heterogeneity (*I*^2^ = 93.80%) (Figure 6).

Egger’s test (intercept = −1.26, 95% CI: −10.09 to 7.58, *p* = 0.74) indicates no evidence of publication bias. Begg’s test (Kendall’s Tau with continuity correlation= −1.00, z = −2.04, *p* = 0.04), however, suggests possible publication bias. In the subgroup analysis, percentage reduction of PYD concentrations was noted in subjects taking Isof extract (−29.16%, 95% CI: −39.58 to −18.73, *p* = 0.00) and Isof in doses of <80 mg per day (−32.04%, 95% CI: −40.42 to −23.66, *p* = 0.00) (Table 2).

Multivariable meta-regression with the covariates of the age of participants (β = 28.21, 95% CI: 4.99–51.42, *p* = 0.02), BMI (β = −22.85, 95% CI: −30.54 to −15.16, *p* < 0.001), and a dose of Isof (β = −33.52, 95% CI −54.18 to −12.86, *p* = 0.001) showed that these covariates had a significant impact on PYD. In contrast, study follow-up period (β = −8.40, 95% CI: −27.92 to 11.12, *p* = 0.40), postmenopausal status (β = 12.40, 95% CI: −10.54 to 35.33, *p* = 0.29), and supplement type were found to have no significant influence on these markers.

**Figure 7:**
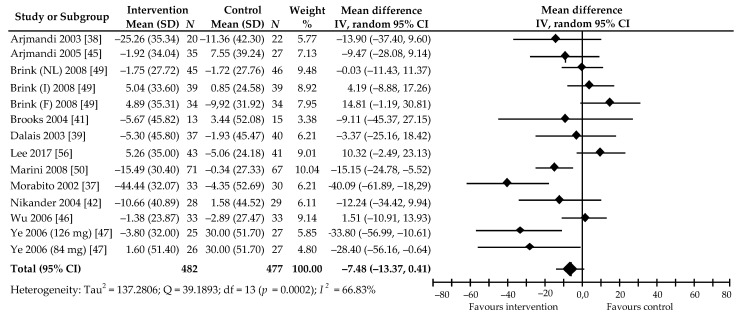
Percentage change in urine DPD values associated with intake of soy isoflavones compared with placebo. Note: in parentheses after the author’s name, Isof dose in studies with more than one treatment arm, or abbreviations of the names of the countries where the study was conducted (NL, the Netherlands; I, Italy; F, France), are shown; data calculated from the random-effects model are presented as weighted mean difference; IV, inverse variance; df, degrees of freedom; the horizontal lines denote the 95% CIs.

We also analyzed the effects of soy Isof supplementation at a daily dose of 85.2 (41.9–126) mg for 3–24 months in 932 postmenopausal women on DPD value. This was based on 14 comparisons from 11 trials [37,38,39,41,42,45,46,47,49,50,56]. In 10 of these, the percentage value of the marker was decreased in the active group; in four, the reduction was statistically significant [37,47,50] (as compared with corresponding control group), while, in another four, it was not significantly increased [46,49,56]. The meta-analysis of all the included studies revealed only a decreasing trend in DPD level: −7.48% (95% CI: −15.37 to 0.41), although this was not statistically significant (*p* = 0.06) (Figure 7). Relatively moderate heterogeneity was also shown (*I*^2^ = 66.83%).

Begg’s test (Kendall’s Tau with continuity correction = −0.27, z = −1.39, *p* = 0.17) and Egger’s test (intercept = −1.84, 95% CI: −4.67 to 0.99, t = −1.41, *p* = 0.18) of publication bias reveal that this is not demonstrated. In the subgroup analysis, a reduction in DPD was significant in women with late post-menopause (>5 y) (−16.49%, *p* = 0.04) and when taking soy Isof extract (−14.63%, *p* = 0.03) (Table 2). A multivariable meta-regression with the covariates of follow-up period (β = 10.75, 95% CI: 0.78–20.72, *p* = 0.03), postmenopausal status (β = −24.75, 95% CI: −35.52 to −13.80, *p* < 0.001), and supplement type (β = −22.51, 95% CI: −33.66 to −11.35, *p* < 0.001) shows that these covariates had a significant impact on DPD. In contrast, age of participants (β = 4.32, 95% CI: −6.85 to 15.50, *p* = 0.45), BMI (β = −3.09, 95% CI: −17.15 to 10.98, *p* = 0.67), and dose of Isof (β = 6.83, 95% CI: −2.56 to 16.23, *p* = 0.15) were found to have no significant influence on these markers.

**Figure 8:**
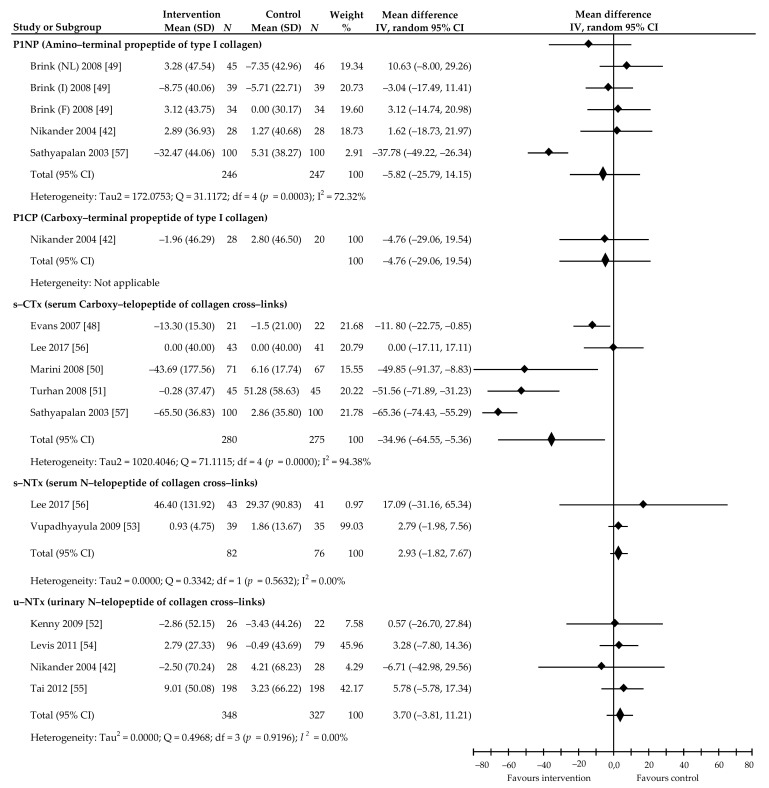
Percentage changes in P1NP, P1CP, serum CTx, serum NTx, and urinary NTx values associated with intake of soy isoflavones compared with placebo. Note: abbreviations of the names of the countries where the study was conducted (NL, the Netherlands; I, Italy; F, France) are in brackets following the name of the lead investigator; data calculated from the random-effects model are presented as weighted mean difference; IV, inverse variance; df, degrees of freedom; the horizontal lines denote the 95% CIs.

Additionally, we searched and analyzed bone turnover markers that were not yet widely used in assessing the effect of soy Isof on bone metabolism. Of the bone formation markers, PINP serum concentrations were tested in five comparisons from three trials [42,49,57]. Here, a statistically insignificant effect of supplement administration was found (−5.82%, 95% CI: −25.79 to 14.15, *p* = 0.56, *I*^2^ = 72.32%). In addition, the result based on one study [42] did not confirm a significant effect of soy Isof on PICP (−4.76%, 95% CI: −29.06 to 19.54, *p* = 0.70). In turn, four trials [42,52,54,55] and two trials [53,56] provided data regarding the impact of soy Isof on resorption markers: urinary NTX and serum NTX, respectively. The analysis did not confirm statistically significant intervention effectiveness, as the summary of results was 3.70%, 95% CI: −3.81 to 11.21, *p* = 0.33, *I*^2^ = 0.00% and 2.93%, 95% CI: −1.82 to 7.67, *p* = 0.22, *I*^2^ = 0.005%, respectively. In turn, summarizing the results of five studies [45,47,48,53,54] revealed significant effects of soy Isof on serum CTX value: −34.96%, 95% CI: −64.55 to −5.36, *p* = 0.02, *I*^2^ = 94.38%. A complete analysis of the abovementioned markers is presented in Figure 8.

## 4. Discussion

In this systematic review and meta-analysis, we incorporated evidence from the most recent studies. Our work indicated that soy Isof supplements did not significantly increase levels of OC (4.16%, *p* = 0.49) and BAP (5.50%, *p* = 0.25), and did not significantly lower the levels of PYD (−12.09, *p* = 0.07) and DPD (−7.48%, *p* = 0.06) in postmenopausal women. Although the results showed no statistical significance, our meta-analysis suggests that soy Isof supplements might be able to decrease levels in bone resorption markers, which may result in increased BMD and a decreased risk of fractures in postmenopausal women. A less pronounced increase in bone formation markers may, however, be due to bone remodeling processes, as changes in their levels occur later than do changes in bone resorption markers [20]. We also performed a sensitivity analysis by excluding one or more studies from the analysis to see how this affected the overall results. This analysis showed that the pooled effects of soy Isof consumption on the considered outcomes did not change substantially if a single study or a few studies were omitted (data not shown). The interpretation of differences in the effect of Isof on bone metabolism markers between the analyzed subgroups based on cofactors may be limited by the relatively low number of RCTs in each subgroup.

The findings of our systematic review and meta-analysis are partially compatible with the results from previously meta-analyses, suggesting insignificant changes to biochemical markers after the administration of soy Isof in postmenopausal women, as compared to those from a control group. The disparity in the effect sizes between earlier meta-analyzes and our analysis may probably be due to our use of the results of the latest published research. Such a dissimilarity may also be associated with differences in the inclusion criteria; our meta-analysis was restricted to studies with follow-up durations that were longer than 3 months.

The meta-analysis performed by Ma et al. [59], based on data from ten RCTS [37,38,39,41,42,43,60,61,62] published between 2002 and 2005, indicated that consumption of mean 68 (39.3–118) mg/day of soy Isof for a period of 4 to 48 weeks caused a significant decrease in DPD value by −2.08 nmol/mmol, 95% CI: −3.82 to −0.34 nmol/mmol, *p* < 0.05. Additionally, an analysis of data from five RCTs [37,38,41,42,45] revealed that the intake of 72 (41.9 to 114) mg of Isof per day for 12 to 48 weeks significantly increased BAP by 1.48 µg/L, 95% CI: 0.22 to 2.75 µg/L, *p* < 0.05. A recently published meta-analysis of Taku et al. [63] summarized the results of qualified works published between 2001 and 2009. These included: ten [37,39,41,42,46,47,50,60,61,62], ten [37,41,42,43,44,46,47,49,50,52], and eight [36,37,44,46,47,60,64,65] studies that concerned DPD, BSAP, and OC, respectively. Analysis revealed that daily ingestion of an average of 56 (14 to 114) mg soy Isof for 10 weeks to 12 months significantly decreased DPD by −18.0%, 95% CI: −28.4 to −7.6, *p* = 0.0007. In turn, daily consumption of an average of 84 (42–114) mg Isof for 3 to 12 months non-significantly increased BAP 8.0%, 95% CI: −4.2 to 20.2, *p* = 0.20. In addition, an insignificant increase of 10.3%, 95% CI: −3.1 to 23.7, *p* = 0.13 in OC was found after ingestion of an average of 73 (38–110) mg Isof per day for a period of 6 weeks to 12 months. Another meta-analysis by Wei et al. [66], comprising eight comparisons from seven studies [39,41,46,47,49,61,67] published between 2002 and 2008, also suggests that administration of Isof in a dose of 69.9 mg/d (22.7–126 mg) for a period of 4 weeks to 12 months results in a significant decrease in DPD value by −23%, 95% CI: −44 to −2, *p* = 0.03. These authors also demonstrated, based on six studies [46,47,49,50,52,68] with seven comparisons, a non-significant reduction in BSAP by −26%, 95% CI: −53 to 1, *p* = 0.06 in women taking 85.1 mg (47–126 mg) of Isof daily for 6–12 months.

An important study concerning the same problems was reported by Arcoraci et al. [69]. The authors performed a post hoc analysis of a previously published study investigating the effects of genistein in postmenopausal women with low bone mineral density. In that study, 59 women received placebo and 62 women received genistein. It turned out that, in the group of women taking genistein, mean bone mineral density increased (from 0.62 g/cm^2^ from the beginning of the study to 0.68 g/cm^2^ after 1 year and 0.70 g/cm^2^ after 2 years), while, in in the placebo group, bone mineral density decreased (from 0.61 g/cm^2^ at baseline to 0.60 g/cm^2^ after 1 year and 0.57 g/cm^2^ after 2 years). The number of women with osteoporosis decreased significantly in the genistein group (from 62 to 18). This suggested that genistein may be useful in the treatment of osteoporosis.

It is difficult to draw final conclusions regarding the impact of soy Isof on other markers of bone turnover (PINP, PICP and NTX in serum and urine), due to the small amount of research devoted to this topic. However, summarizing the results of five studies [48,50,51,56,57] revealed significant effects of soy Isof supplements on CTX values: −34.96%, *p* = 0.02. Taku et al. [63], in their meta-analysis, also signaled this effect, albeit without attempting to summarize the results of published studies.

### 4.1. Limitations of this Study

There are several limitations that need to be mentioned and that should be considered when interpreting the data. The results of the meta-analysis are based on a relatively limited number of available studies, and on a different number of participants and variable observation time in individual samples. This may result in insufficient statistical power and limit the drawing of final conclusions. The lack of standardized research protocols, including the variable content of Isof in preparations (methylated forms, glycosides and aglycones) and their different dosing, as well as the impact of other foods consumed during the study, may make it difficult to compare the obtained data. Furthermore, the characteristics of women participating in the study may also have an impact; various comorbidities, years since menopause, as well as inter-individual differences in the metabolism and bioavailability of Isof (different status of equole producers) may cause variability in the response to its use. Other limitations are related to the fact that the analyzed works may not represent all research related to this topic, especially those published in a language other than English.

### 4.2. Mechanisms of Action of Soy Isoflavones

There are numerous proposed mechanisms by which soy Isof modulates bone metabolism. Soy Isof has a characteristic chemical structure (its ring B is connected to the C3 position of the C ring instead of the C2 position) that is reminiscent of the structure of estrogens, particularly estradiol 17-β, and exhibits weak estrogenic activity by binding to estrogen receptors (ER), with a higher affinity towards ER-β [70,71]. Similar to estrogen, soy Isof promotes osteoblast differentiation via ERs [72,73]. Other possible mechanisms may be its effects on the intensity of the secretion of osteoprotegerin (OPG) by osteoblastic cells. This could decrease the interactions of receptor activator for nuclear factor κ B ligand (RANKL), thereby suppressing osteoclastic activities, as the receptor activator for nuclear factor κ B (RANK) is located on the osteoclast surface [74,75]. There are suggestions that Isofs, especially genistein and daidzein, are capable of activating nuclear peroxisome proliferator activated receptors (PPAR) and mediating PPAR gene expression to modulate osteoclast function [76,77]. In addition, soy Isof can cause reduction in bone turnover and increased osteoblastic activity through inhibition of both tumor necrosis factor-α (TNF-α) and interleukin-2 (IL-2) [78]. Furthermore, there are reports that soy Isof may engender an osteoprotective effect by: inhibiting the increase in parathyroid hormone (PTH) levels [79]; regulating nitric oxide (NO) production [80]; inhibiting tyrosine kinase activity [81]; activating adenosine monophosphate-activated protein kinase (AMPK) [82,83]; stimulating the insulin-like growth factor-1 (IGF-1) [84,85]; upregulating of transforming growth factor-β (TGF-β) [86]; activating receptors for vitamin D3 [87]; or antioxidant activity [88]. Finally, it is worth mentioning that earlier studies suggested that the soy isoflavones and soy protein supplementation affect endometrial thickness, induce endometrial hyperplasia, and increase endometrial cancer risk [89,90]. However, recent scientific reports do not confirm the previous observations [91,92,93].

## 5. Conclusions

The meta-analysis of the included studies revealed some statistically insignificant observations indicating that soy isoflavones intake is associated with a trend of increased levels of OC and BAP, as well as a trend in reduced levels of PYD and DPD. However, it is difficult to draw far-reaching conclusions based on these results due to the lack of statistical significance. Multivariable meta-regression showed that some covariates had a significant impact on individual bone markers. These include: supplement type on OC value, age of participants, follow-up period, BMI, dose of Isof and supplement on BAP, age of participants, BMI and dose of Isof on PYR, and follow-up period, postmenopausal status, and supplement type on DPD. Based on the literature, we conclude that women using soy Isof supplements for menopausal symptoms may gain additional benefits from a specific effect on healthy bone, but this requires further research. It should be taken into account that not all women show rapid bone loss after menopause, and measuring values of biochemical markers of bone turnover is clinically important for identifying women at high risk of future fractures. Soy isoflavones may have a beneficial effect on bone formation markers, but this requires an extensive multi-center research analysis.

## Figures and Tables

**Table 1:** Characteristics of selected randomized controlled studies assessing the effects of soy isoflavones on biochemical bone turnover markers in postmenopausal women.

First Author Year Country	Study Design Follow-up	Participants ^a^ Age, y (Range) Treated/Control	Intervention form Therapy and Daily Dosage ^b^ vs. Control	Indices ^c^ Formation Markers Resorption Markers
Knight [36] 2001 UK	Double-blind, parallel group 3-months	53.1 ± 4.6 (40–65) 3.6 ± 4.5 ysm 9/11	Beverage powder 60 g SP, 134.4 mg Isof (aglycones: 77.4 mg) vs. casein protein	OC
Morabito [37] 2002 Italy	Double-blind, parallel group 12-months	51.5 ± 4.5 (47–57) 6.5 ± 2.5 ysm BMI 24 ± 3 T-score = 1 SD 30/30	Tablet 54 mg Isof (Gen, purity ~98%) vs. placebo	OC, BAP YD, DPD
Arjmandi [38] 2003 USA	Double-blind, parallel group 3-months	62.1 ± 11.0 BMI 32.2 ± 8.0 20/22	Powder in drink mix 40 g SP, 88.4 mg Isof vs. 40 g milk protein	BAP DPD
Dalais [39] 2003 Australia	Double-blind, parallel group 3-months	60.0 ± 6.2 (50–75) BMI 25.5 ± 4.6 38/40	Powder mixed with drinks or food 40 g SP, 118 mg Isof (aglycones: 69 mg) vs. 40 g casein protein	PYD, DPD
Uesugi [40] 2003 Japan	Double-blind, parallel group 3-months	53.7 ± 6.9 (45–65) 6.0 ± 6.0 ysm BMI 22.6 ± 2.8 11/10	Capsule 61.8 mg Isof (glycosides: 31.2 mg Dai, 6.8 mg Gen, 21.6 Gly) vs. placebo (dextrin)	PYD
Brooks [41] 2004 Canada	Double-blind, parallel group 16-weeks	53.4 ± 3.02 <5 ysm BMI 27.4 ± 5.3 13/15	Muffins 25 g SP, 41.9 mg Isof (15.5 mg Dai, 25.7 mg Gen, 0.7 mg Gly) vs. 25 g whole-wheat flour	BAP DPD
Nikander [42] 2004 Finland	Double-blind, cross-over trial 3-months	55 ± 6 (35–69) ≥ 5 ysm BMI 26.3 ± 3.3 28/28	Tablet 114 mg Isof (aglycones: 58% Gly, 36% Dai,6% Gen) vs. placebo	BAP, PINP, PICP PYD, DPD, NTX
Kreijkamp-Kaspers [43] 2004 Netherlands	Double-blind, parallel group 12-months	66.6 ± 4.8 (60–75) 18.0 ± 7.0 ysm BMI 26.2 ± 3.8 88/87	Powder mixed with drinks or food 25.6 g SP: 99 mg Isof (aglycones: 52 mg Gen, 41 mg Dai, 6 mg Gly) vs. 25.6 g milk protein	BAP
Harkness [44] 2004 USA	Double-blind, cross-over trial 6-months	70.6 ± 6.3 19.1 ± 5.5 ysm BMI 26.1 ± 4.8 T = score ≥ −2.5 10/9	Pill 110 mg Isof (98% glycosides and 2% aglycones) vs. placebo	OC, BAP
Arjmandi [45] 2005 USA	Double-blind, parallel group 12-months	54.5 ± 5.5 (<65) 5.5 ± 5.0 ysm BMI 27.9 ± 5.3 35/27	Snack, drink mix or cereal 25 g SP, 60 mg Isof vs. 20 g protein (devoid of soy and isof)	OC, BAP DPD
Wu [46] 2006 Japan	Double-blind, parallel group 6-months	54.3 ± 2.9 (45–60) 3.2 ± 1.7 ysm BMI 21.1 ± 2.4 33/33	Capsule 75 mg Isof (47 mg aglycones) vs. placebo (dextrin)	OC, BAP DPD
Ye [47] 2006 China	Single-blind, parallel group, 3 arms trial 6-months	52.3 ± 3.3 (45–60) 2.6 ± 1.5 ysm BMI 22.6 ± 2.3 25/26/27	Capsule (a) 126 mg Isof, (b) 84 mg isoflavones (aglycones: 52% Dai, 15% Gen, 33% Gly) vs. placebo (starch)	OC, BAP DPD
Evans [48] 2007 USA	Double-blind, parallel group 9-months	63.1 ± 5.1 (50–65) 8.2 ± 5.1 ysm BMI 26.8 ± 2.8 21/22	Protein beverage products 25.6 g SP, 91.5 mg Isof (aglycones) vs. 25.6 g milk protein	BAP CTX
Brink [49] 2008 Netherlands, Italy, France	Double-blind, parallel group, international, multicenter trial 12-months	53 ± 3 2.7 ± 1.3 ysm BMI 24.5 ± 2.1 118 (NL 45; I, 39; F, 34)/119 (NL, 46; I, 39; F, 34)	Biscuits or bars 110 mg Isof (aglycones: 60–75% Gen, 25–35% Dai, 1–5% Gly) vs. control	BAP, PINP PYD, DPD
Marini [50] 2008 Italy	Double-blind, parallel group 24-months	53.7 ± 2.5 3.6 ± 2.6 ysm BMI 24.9 ± 3.7 71/67	Tablet 54 mg Isof (Gen, purity ~98% vs. placebo	BAP PYD, DPD, CTX
Turhan [51] 2008 Turkey	Single-blind, parallel group 6-months	51.5 ± 5.2 (42–59) 3.7 ± 1.8 ysm BMI 27.0 ± 3.1 45/45	Tablet 80 mg isof (aglycones: 59.6 mg Gen, 15.6 mg Dai, 4.8 Gly) vs. placebo (starch)	OC CTX
Kenny [52] 2009 United States	Double-blind, parallel group 12-months	72.9 ± 5.9 (60–93) 23.1 ± 9.0 ysm BMI 28.9 ± 5.9 25/22	Dietary protein + pill 8 g SP + 105 mg Isof (aglycones) vs. 18 g mix of protein (whey, caseinate, egg white) + placebo (maltodextrin)	BAP NTX
Vupadhyayula [53] 2009 USA	Double-blind, parallel group 24-monts	66.5 ± 4.5 (>55) 14.3 ± 5.4 ysm BMI 26.3 ± 3.8 T-score > −2.5 30/35	Powder 25 g SP, 90 mg Isof vs. 25 g milk protein	NTX
Levis [54] 2011 USA	Double-blind, parallel group 24-months	52.5 ± 3.3 (45–60) BMI 26.3 ± 3.3 T-score > −2.0 99/83	Tablet 200 mg Isof (aglycones: 91 mg Gen, 103 mg Dai) vs. placebo	NTX
Tai [55] 2012 Taiwan	Double-blind, parallel group 24-months	55.9 ± 3.8 (45–65) 5.1 ± 2.7 ysm BMI 22.9 ± 2.6 200/199	Capsule 300 mg Isof (aglycones: 57.5% Gen, 42.5% Dai) vs. placebo (cellulose)	BAP NTX
Lee [56] 2017 Korea	Double-blind, parallel group 3-months	53.6 ± 3.4 (45–60) 3.6 ± 2.3 ysm BMI 19.0–30.0 41/43	Tablet 70 mg Isof (glycosides: 38 mg Gly, 20 mg Dai, 12.4 mg Gen)vs. placebo (dextrin)	OC, BAP DPD, NTX, CTX
Sathyapalan [57] 2017 UK	Double-blind, parallel group 6-months	50 y≤ 2 ysm, BMI, 26.9 ± 5.8 60/60	Snack bar 15 g SP, 66 mg Isof (90% glycosides, 10% aglycones) vs. 15 g soy protein alone	PINP CTX
Zhang [58] 2020 China	Double-blind, parallel group 6-months	58 ± 3.1 (40–55) 4.0 ± 3.2 ysm BMI 23.3 ± 3.2 38/37	Tablet 60 mg Isof vs. placebo (microcrystalline cellulose, dextrin)	OC

Data are presented as mean ± standard deviation. Here: ^a^ BMI, body mass index (kg/m^2^); *n,* number of women; T-score, the standard deviation from peak bone mass in healthy people 30-year-old; ysm, years since menopause; NL, the Netherlands; I, Italy; F, France; ^b^ Agly, aglycones; Dai, daidz(in)ein; Gen, genist(in)ein; Gly, glycit(in)ein; Glyco, glycosides; Isof, isoflavones; SP, soy protein; ^c^ BAP, bone alkaline phosphatase; CTX, C-terminal telopeptide of type I collagen; u-DPD, urine deoxypyridinoline; OC, osteocalcin; PINP, procollagen I amino-terminal propeptide; u-PYD, urine pyridinoline; u-NTX, urine N-terminal telopeptide of type I collagen.

**Table 2:** Pooled estimates of treatment effect on biochemical bone turn-over markers in subgroups of trials.

Subgroup Outcomes	Osteocalcin (OC)					Bone Alkaline Phosphatase (BAP)					Pyridinoline (PYD)					Deoxypyridinoline (DPD)				
	*N*	*n*	WMD (95% CI)	*p*	*I*^2^ (%)	*N*	*n*	WMD (95% CI)	*p*	*I*^2^ (%)	*N*	*n*	WMD (95% CI)	*p*	*I*^2^ (%)	*N*	*n*	WMD (95% CI)	*p*	*I*^2^ (%)
Overall effects	10	581	4.16 (−7.72, 16.04)	0.49	85.35	18	1560	5.50 (−3.81, 14.82)	0.25	92.39	8	589	−12.09 (−25.37,1.20)	0.07	89.12	14	959	−7.48 (−15.37, 0.41)	0.06	66.83
Follow-up period																				
≤6 months	8	459	0.27 (−10.93, 11.47)	0.96	83.68	8	400	2.07 (−1.85, 5.98)	0.30	0.00	3	154	−17.04 (−42.00, 8.31)	0.08	77.94	8	459	−8.56 (−19.56, 2.43)	0.13	56.68
>6 months	2	122	6.47 (−81.55, 94.48)	0.88	83.60	10	1160	8.10 (−7.16, 23.36)	0.30	95.34	5	435	−9.85 (−26.70, 7.00)	0.25	92.08	6	500	−6.48 (−18.82, 5.85)	0.30	78.14
Menopausal status																				
<5 years	7	440	−0.96 (−12.70, 10.78)	0.87	85.31	11	1112	6.97 (−5.83, 19.76)	0.29	94.80	5	431	−4.79 (−18.40, 8.83)	0.49	86.09	10	715	−4.28 (−13.05, 4.48)	0.34	67.53
≥5 years	3	141	14.98 (−24.91, 54.87)	0.46	72.71	7	448	1.90 (−6.80, 10.60)	0.67	58.24	3	158	−25.87 (−52.81, 1.07)	0.06	87.73	4	244	−16.49 (−32.17, −0.81)	0.04	53.73
Age																				
<54 years	6	359	14.18 (−13.96, 42.33)	0.32	90.13	9	652	10.25 (−6.58, 27.08)	0.23	95.44	6	456	−14.45 (−30.70, 1.81)	0.08	91.97	9	655	−8.65 (−20.25, 2.95)	0.14	78.34
≥54 years	4	222	−3.57 (−13.30, 6.16)	0.47	63.70	9	908	0.72 (−3.51, 4.94)	0.74	19.02	2	133	−3.50 (−16.02, 9.03)	0.13	0.00	5	304	−4.87 (−12.95, 3.20)	0.24	0.00
BMI ^§^																				
≤24.9 kg/m^2^	6	390	14.17 (−1.27, 29.61)	0.07	89.01	10	1088	10.18 (−2.88, 23.25)	0.13	95.34	6	456	−14.45 (−30.70, 1.81)	0.08	91.97	9	693	−7.18 (−17.86, 3.50)	0.19	78.85
>24.9 kg/m^2^	3	171	−15.13 (−46.96, 16.69)	0.35	70.69	8	472	−3.09 (−8.31, 2.13)	0.25	0.00	2	133	−3.50 (−16.02, 9.03)	0.58	0.00	5	266	−9.53 (−19.73, 0.68)	0.07	0.00
Intervention type																				
Soy protein	2	82	−16.11 (−44.40, 12.19)	0.26	0.00	9	634	−1.29 (−5.77, 3.20)	0.57	0.00	4	314	2.79 (−2.64, 8.22)	0.31	0.00	7	446	0.53 (−6.03, 7.10)	0.87	5.10
Isoflavone extract	8	499	7,03 (−5.79, 19,84)	0.28	88.93	9	926	10.61 (−4.01, 25.23)	0.15	95.75	4	275	−29.16 (−39.59, −18.73)	0.00	50.68	7	513	−14.63 (−27.88, −1.38)	0.03	77.90
Isoflavone dose																				
<80 mg/day	5	347	8.07 (−7.79, 23.92)	0.32	89.88	6	438	14.97 (−7.65, 37.60)	0.19	94.96	3	219	−32.04 (−40.42, −23.66)	0.00	24.47	6	441	−9.02 (−22.09, 4.05)	0.18	75.61
≥80 mg/day	5	234	3.87 (−23.35, 31.09)	0.78	78.27	12	1122	0.15 (−2.74, 3.04)	0.92	0.00	5	370	1.91 (−3.34, 7.16)	0.48	0.00	8	518	−6.33 (−16.72, 4.06)	0.23	60.38

Abbreviations: CI, confidence interval; *I*^2^, coefficient of inconsistency; *N*, number of comparisons; *n*, number of subjects; *p*, probability value; WMD, weighted mean difference in percentage (%); ^‡^ containing isoflavone; ^§^ data not available for OC according to the work of Knight [36].

## Data Availability

The data that support the findings of this study are available from the corresponding author, upon reasonable request.
